# Glutamate Signaling and Filopodiagenesis of Astrocytoma Cells in Brain Cancers: Survey and Questions

**DOI:** 10.3390/cells11172657

**Published:** 2022-08-26

**Authors:** Mitra Tabatabaee, Frederic Menard

**Affiliations:** 1 Department of Biology, The University of British Columbia, Kelowna, BC V1V 1V7, Canada; 2 Department of Chemistry, The University of British Columbia, Kelowna, BC V1V 1V7, Canada

**Keywords:** astrocytes, astrocytoma, glutamate signaling, morphological response, filopodiagenesis

## Abstract

Astrocytes are non-excitable cells in the CNS that can cause life-threatening astrocytoma tumors when they transform to cancerous cells. Perturbed homeostasis of the neurotransmitter glutamate is associated with astrocytoma tumor onset and progression, but the factors that govern this phenomenon are less known. Herein, we review possible mechanisms by which glutamate may act in facilitating the growth of projections in astrocytic cells. This review discusses the similarities and differences between the morphology of astrocytes and astrocytoma cells, and the role that dysregulation in glutamate and calcium signaling plays in the aberrant morphology of astrocytoma cells. Converging reports suggest that ionotropic glutamate receptors and voltage-gated calcium channels expressed in astrocytes may be responsible for the abnormal filopodiagenesis or process extension leading to astrocytoma cells’ infiltration throughout the brain.

## 1. Introduction

Tumor progression in the brain is different and more aggressive than that in other tissues [1,2]. Indeed, the average life expectancy for patients suffering from high-grade gliomas is only 12–16 months. This short timeline is due to aggressive diffusion, adaptive resistance against chemotherapy, and a high probability of local recurrence after surgery [3]. Cancerous cells arising from glia, astrocytes in particular, are naturally prone to extending cell projections and can infiltrate tissue and spread to distant brain regions. This infiltrative property of transformed astrocytes is thought to be one mechanism by which astrocytoma cells resist chemo- and radiotherapy treatments, or reoccur after ablative surgery [4,5,6,7]. Identifying the stimuli and receptors that contribute to the aberrant morphology of astrocytoma cells might offer means to intervene, slow, or prevent brain tumor infiltration, thereby decreasing the odds of tumor recurrence post-treatment. While several research efforts aim to shed light on how undesired filopodiagenesis occurs, no consensus has been reached yet.

**Figure 1:**
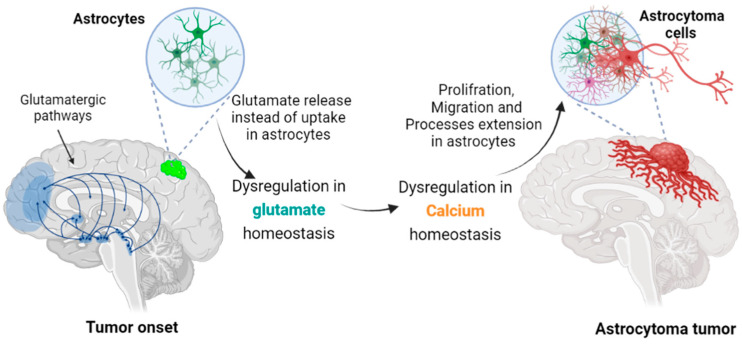
Transformation of astrocytes leading to astrocytoma cells. In grade II astrocytoma, the mutated astrocytes extend cellular projections that can infiltrate tissue distant from their soma location. Aberrant, excessive glutamate release at synapses is neurotoxic and could allow a tumor cell to spread in the interstitial space liberated by the retracting neurons. An associated glutamate-induced intracellular Ca^2+^ rise in astrocytoma could also accelerate filopodiagenesis and cancer cell migration.

This review discusses possible mechanisms that govern astrocytoma cell tissue infiltration. It outlines how glutamate dysregulation in the brain may be responsible for the aberrant morphology of transformed astrocytes (Figure 1). The effect of glutamate signaling on the morphology of normal astrocytes versus astrocytoma cells is presented first. This is followed by the discussion of glutamate receptors as candidate initiators of undesired glutamate-dependent morphology such as filopodiagenesis, and the involvement of voltage-gated calcium channels via their associated glutamate-sensitive α2δ subunit. Each section presents the context for current hypotheses, along with knowledge gaps or open questions remaining to be addressed.

### 1.1. Tumors, Glioma and Astrocytoma

Gliomas are the most prevalent primary tumors in the central nervous system [7,8]. The innate ability of glioma cells to use their long processes for invasion, proliferation, and replication, and to enable long-range communication within a network of cancer cells is emerging [4,5,6]. Gliomas are classified according to their parent normal glia: astrocytic tumors, oligodendroglial tumors, oligoastrocytoma tumors, ependymal tumors, and neuronal and mixed neuronal–glial tumors (e.g., ganglioglioma and glioblastoma multiforme) [9,10]. Gliomas are also classified according to their invasiveness as the major determinant of malignancy, starting with grade I tumors that have low proliferative potential and are usually circumscribed to a single region. More infiltrating malignant glioma tumors are classified as grade II (astrocytoma and oligodendroglioma), grade III (anaplastic oligodendroglioma, anaplastic astrocytoma, anaplastic oligoastrocytoma, and anaplastic ependymoma), and grade IV (glioblastoma). [3,7,8]. Grade II astrocytoma cancers are useful models for gaining important biochemical insight, as they arise from transformed astrocytes [11].

### 1.2. Dynamic Morphology of Astrocytes

Astrocytes extend and retract their fine processes at synaptic contacts [12,13,14,15]. Such dynamic reshaping is influenced by their neighboring cells and the architecture of each brain region [3,4,5,6]. For instance, live confocal imaging of the hippocampus, brainstem, and cortex has demonstrated that astrocytes frequently probe at glutamatergic synapses to eventually enwrap them tightly [16,17,18]. Peripheral astrocytic processes (PAPs) in healthy cells show a clear directional motility preference toward glutamatergic synapses [19,20,21,22]. This suggests that they respond to chemical signals released externally at the synaptic cleft, specifically the neurotransmitter glutamate [23,24,25]. While the malignancy and therapy resistance of astrocytoma tumors correlate strongly with the abnormal morphology of astrocytoma cells, e.g., extra-long processes and excessive network elaboration, the exact cause is still unclear. Dysregulation of glutamate homeostasis has been suggested as a plausible mechanism for the overactive elongation of processes in astrocytoma cells [4,5,6,26].

### 1.3. Morphology of Astrocytoma Cells

In contrast to normal astrocytes, astrocytoma cells develop longer, more dynamic and highly elaborated processes [4,5,27,28]. The extension and retraction of their processes are not restricted to synaptic sites; they can infiltrate tissue and create networks that extend beyond their initial location [29,30]. In a tumor, these spreading cells create a fibrillary background that can keep their microenvironment shielded from interventions or treatments such as chemo- and radiotherapy. This infiltration of long filopodia within tissue is what allows a malignant glioma tumor to expand throughout the brain more rapidly than other cancer types [26,31,32].

Understanding how to prevent uncontrolled filopodiagenesis in astrocytoma is an attractive, logical step toward stopping tumor progression. These cells most likely use the same molecular mechanisms as their parent astrocytes, but dysregulation in chemical signaling seems to cause their filopodia to extend abnormally. Filopodia are long, cylindrical cell projections filled with bundles of parallel actin filaments [30]. They occur in several cell types and often act as pathfinders in response to guiding chemical cues [31,32,33,34]. Filopodiagenesis depends on the intracellular release of Ca^2+^ ions, and it arises from the reorganization of sheet-like actin arrays. The molecular details that underpin the initiation and maintenance of filopodia are just beginning to emerge [33,34,35,36]. The proteins involved in the guided chemical responses of the and/or their structural reorganization have attracted attention. In astrocytoma cells, such promising candidates are glutamate-sensitive ion channels.

## 2. Glutamate Signaling

**Figure 2:**
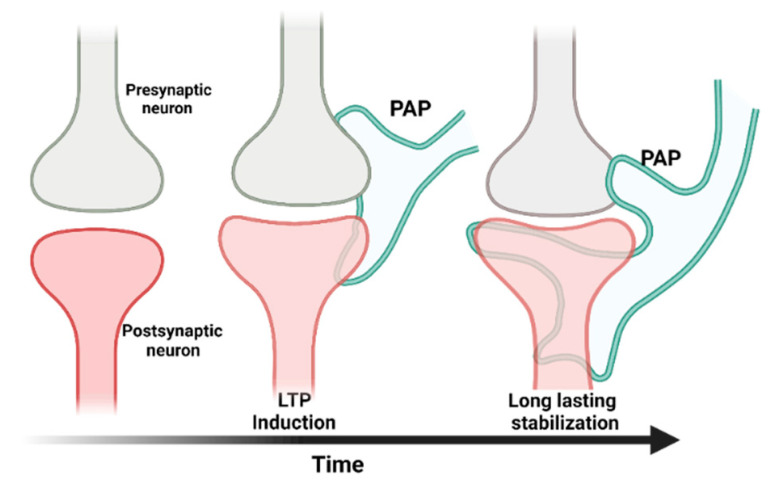
The tripartite synapse model. A tripartite synapse consists of a pre-synapse (gray), a post-synapse (red), and a perisynaptic astrocyte process (PAP, green). PAPs undergo structural changes in response to long-term potentiation. The growth of post-synaptic dendritic spines is associated with enhanced motility of PAPs, which increases their coverage of synapses and strengthens the synaptic connection [38].

Glutamate is the primary mediator of excitatory signals in the CNS; it can be released and sensed by both neurons and astrocytes [37,38,39]. More specifically, astrocytes express several receptors and enzymes that are essential for maintaining glutamate homeostasis at a synaptic cleft [40,41,42,43,44,45]. In healthy synaptic communication, each neuronal synapse is usually enveloped by an astrocytic projection to form a tripartite synapse (Figure 2) [37,43]. The morphology of these ensheathing astrocytic projections is known to be sensitive to glutamate [23,43,46]. In glioma, the infiltrative activity of cancer cells is associated with filopodium extension and elevated glutamate levels. How the phenomenon takes place is still unclear, but several hypotheses point to calcium-dependent events.

**Figure 3:**
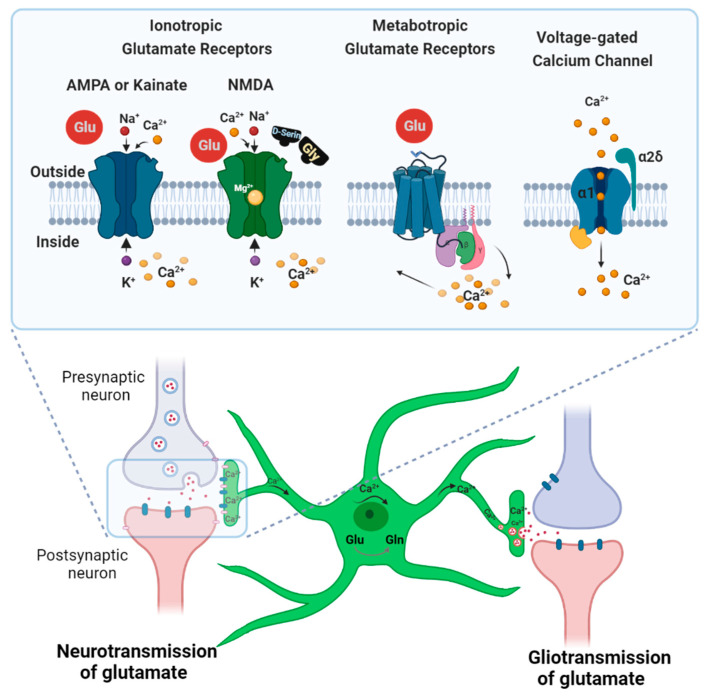
Scheme outlining glutamate and calcium signaling in astrocytes at a glutamatergic synapse. Astrocytes respond to glutamate stimulation with a strong calcium influx, followed by process extension. Such transient increases in Ca^2+^ concentration in astrocytes can also cause gliotransmitter release. To prevent excitotoxicity, astrocytes also take up glutamate to recycle it into non-toxic glutamine [49]. Ionotropic (AMPA, KA, and NMDA) and metabotropic glutamate receptors contribute to the intracellular Ca^2+^ rise in neurons and astrocytes. Voltage-gated calcium channels are also expressed at synapses in astrocytes and play a role in cellular calcium events.

When astrocytes sense a glutamate signal, it is followed by a rapid rise in the intracellular Ca^2+^ ion concentration, which is required for filopodiagenesis (Figure 3) [40,41,47,48]. Thus, the glutamate receptors that act as Ca^2+^ ion channels or release Ca^2+^ from internal sources are prime suspects as potential contributors to the aberrant morphology of astrocytoma cells. The following sections discuss ion channels that may be responsible for the uncontrolled process extension of astrocytoma cells.

### Glutamate Receptors in Astrocytes and Astrocytoma Cells

In astrocytes, the receptors that contribute to Ca^2+^ events following glutamate stimulation are heterogeneous; their expression profiles vary according to the regions of the brain and stages of development [16,17,48,50]. The two main families of glutamate-sensitive receptors are the ionotropic and metabotropic glutamate receptors (iGluR and mGluR, respectively). iGluRs are ligand-gated ion channels that mediate excitatory neurotransmission upon the binding of glutamate [50,51]. They are well-studied in neurons but not in astrocytes. Three iGluR subfamilies have been found in astrocytes: α-amino-3-hydroxy-5-methyl-4-isoxazolepropionic acid receptors (AMPARs), kainic acid receptors (KARs), and *N*-methyl-D-aspartate receptors (NMDARs) [52,53,54]. AMPA and KA receptors are usually considered together as non-NMDA receptors due to their structural similarity and their lack of selective antagonists that would allow unequivocal distinction [55,56,57].

Calcium-permeable AMPARs have been identified in glioma cells as well as in astrocytes from the forebrain, the neocortex, and Bergmann glial cells of the cerebellar cortex [43,46]. They have been shown to be involved in glutamate-mediated proliferative signals in glioma cells [11,58]. It is still unknown whether they also contribute to the morphological response of astrocytoma cells upon exposure to glutamate.

Astrocytes and astrocytoma cells also express kainic acid receptors (KARs), but their function remains largely unexplored [23,58,59]. Since astrocytoma progression is known to be affected by perturbed glutamate homeostasis [60,61,62,63], our lab investigated the relationship between the glutamate stimulation of astrocytoma cells and their aberrant morphology [64,65]. Glutamate was found to trigger the rapid extension of processes in astrocytoma cells (as observed in their parent astrocytes). We also found that glutamate receptors sensitive to kainic acid (GluK) are involved in filopodiagenesis [23,24,43]. This selective activation of KARs was sufficient to cause filopodium extension in astrocytoma cells, suggesting for the first time that KARs play a direct role in glutamate-induced filopodiagenesis.

Some astrocytoma cells have been reported to express subunits of NMDARs; however, the absence of the NR1 subunit makes these proteins unfunctional [58]. Despite having been identified in normal astrocytes from a few regions of the brain, NMDAR’s activity is barely detectable due to the high polarization of the astrocytes’ membranes (resting potential: –80 mV) [16,17,44,63,66]. Only in cultured cortical astrocytes have active NMDARs been observed [67].

Astrocytes stimulated with glutamate show a sharp rise in intracellular Ca^2+^ concentration rise that depends on metabotropic glutamate receptor 5 (mGluR5) [68,69]. These G protein-coupled receptors release calcium from internal stores via an IP_3_-mediated pathway by activating Gq and phospholipase C [42,45,68,69]. However, whether mGluR5 is essential for astrocytic glutamate signaling is still an unanswered question, as literature reports vary. For instance, glutamate-dependent calcium signaling was found to involve mGluR5 in juvenile hippocampal astrocytes, but mGluR5 agonists failed to induce the same calcium cascade in astrocyte soma from the adult brain [43,48]. Similarly, glutamate released by mossy fibers of the mature hippocampus was shown to induce only partial calcium signals via mGluR5 in astrocytes [70,71].

Thus far, ionotropic glutamate receptors sensitive to AMPA and KA have shown the most direct correlation with triggering filopodiagenesis in astrocytoma cells. It should be noted that astrocytomas also express mGluRs whose function remains unidentified [58].

## 3. Calcium Influx in Astrocytoma

The presence and role of calcium influx in astrocytes have often been debated over the past decades, but the phenomenon is now generally accepted [72,73]. Consequently, modifying the calcium influx in glioma and astrocytoma is viewed as a potential therapeutic avenue for treating brain cancers [74]. The proteins that govern the calcium influx in astrocytoma have not been unambiguously established. However, the repeated observation of glutamate-dependent calcium signaling events presents a possible means by which to stifle astrocytoma progression.

### 3.1. Glutamate Signaling vs. Intracellular Calcium

Instead of sequestering and recycling excess glutamate like normal astrocytes, astrocytoma cells increase the glutamate concentration at synaptic contacts [3,60,61,62,63,75,76]. This glutamate accumulation can be caused by the lack of Na^+^-dependent glutamate uptake [75,77] or by the hyperactive release of glutamate [37,43,61]. Such a local excess of neurotransmitters leads to a Ca^2+^ rise in astrocytoma that creates a positive feedback loop and amplifies glutamate release [3,75]. The resulting overstimulation of neurons is excitotoxic—the associated uncontrolled intracellular Ca^2+^ can cause their apoptosis [75,78,79,80].

The glutamate receptors responsible for the large Ca^2+^ rise in astrocytoma cells have not yet been identified. As described above, we recently reported that glutamate receptors sensitive to kainic acid are involved in filopodiagenesis. Intriguingly, we also found that Ca_V_1.2 voltage-gated calcium ion channels may also participate in filopodiagenesis in astrocytoma cells [64,65,81,82].

### 3.2. Glutamate Signaling and Votage-Gated Calcium Channels

The possibility that external glutamate stimulation can lead to the activation of Ca_V_ channels in astrocytoma raises several mechanistic questions that are only starting to be investigated. In the CNS, high-voltage Ca_V_s were thought to be exclusive to excitable cells such as neurons (i.e., the Ca_V_1 calcium channel family) [83], but they are now known to also be expressed in non-excitable cells such as astrocytes and astrocytoma cells [84,85]. Functional Ca_V_s are composed of a pore-forming α1 subunit that conducts Ca^2+^ ions, an internal β subunit, and an extracellular α2δ subunit [76].

The function of Ca_V_1s is not limited to excitability in neurons: they help to regulate ion homeostasis [86,87,88], and in non-excitable cells, Ca_V_1s were shown to contribute to non-electrical events that require a high volume of Ca^2+^ input, such as actin cytoskeleton reorganization [33,34,89]. For instance, calcium “hotspots” arising from Ca_V_1 clusters promote morphological changes in neuronal growth cones [90,91,92].

The greater frequency of opening Ca_V_ channels observed during filopodiagenesis suggests their involvement in filopodium formation [33]. Indeed, calcium signaling has been shown to regulate filopodiagenesis via a network of signaling proteins such as Ca^2+^-activated K^+^ channels (BK) coupled to voltage-gated calcium channels (Ca_V_s) [33,93]. Moreover, pharmacological Ca_V_ blockers have been shown to reduce glioma cell proliferation and infiltration in the brain [94]. Paradoxically, Ca_V_ agonists did not enhance tumor growth and did not increase intracellular Ca^2+^ concentrations [95,96,97,98].

While studying glutamate signaling, our team observed a Ca_V_-channel-dependent morphological response in astrocytoma cells [82]. The stimulation of U118 astrocytoma cells with glutamate caused rapid filopodiagenesis. Selectively blocking the pore-forming α1 subunit of Ca_V_s only reduced the extension of processes. In contrast, using antagonists selective for the extracellular α2δ subunit of Ca_V_s completely inhibited filopodiagenesis [65,82]. Moreover, we observed a rapid translocation and redistribution of high-voltage Ca_V_s at the membrane when astrocytoma cells were exposed to glutamate [81]. These observations suggest that the morphological response to glutamate may involve the extracellular α2δ subunit of Ca_V_s in astrocytoma cells. Whether α2δ exerts its effect by modulating Ca^2+^ or other pathways remains to be elucidated. It should be noted that, while the α2δ protein was first discovered with Ca_V_s, reports are emerging of its association with other proteins such as NMDA or BK channels as well [99,100].

The complex interplay between glutamate exposure and Ca_V_ activation has been reported in neurons but not in astrocytes [101]. However, the expression of functional voltage-gated Ca^2+^ channels as well as glutamate receptors in astrocytes and astrocytoma cells has been established [43,82,84,102,103]. Hypotheses are starting to converge to involve undesired filopodiagenesis triggered by the possible binding of excess glutamate to Ca_V_’s α2δ subunit. However, much more work is needed to control for other Ca^2+^-mediated pathways.

## 4. Concluding Remarks

Studies on tumor progression have shown that the abnormal accumulation of extracellular glutamate at the synaptic cleft leads to disrupted Ca^2+^ homeostasis. Most reports studying filopodiagenesis caused by cellular calcium signaling in astrocytoma have focused on glutamate receptors. In contrast, voltage-gated calcium channels have been much less investigated [11,48,62,104]. Empirical data now suggest that the rapid process extension of astrocytoma cells associated with glutamate signaling may involve kainic acid receptors (KARs) and/or Ca_V_1 voltage-gated calcium channels. This arguably simplistic view will likely gain complexity as more data become available.

Several questions remain to be answered on what governs the astrocytic morphological changes induced by glutamate signaling. For instance, what is the function of kainic acid receptors (KARs) expressed in astrocytes and astrocytoma cells? Are there cases where AMPA receptors initiate filopodiagenesis? What is the role of other mGluRs expressed in astrocytoma cells? How do Ca_V_s influence glioma tumor growth? How does the extracellular α2δ subunit of Ca_V_s contribute to the morphological response in astrocytoma? Does α2δ exert its effect by modulating Ca^2+^ directly, or by other pathways? Are there more glutamate receptors responsible for the uncontrolled Ca^2+^ rise in astrocytoma cells?

Taken together, the accumulating evidence for a glutamate-dependent morphological response of astrocytoma cells points to new research avenues for the role of glutamate signaling in the progression and malignancy of astrocytoma tumors. This review aimed to gather, in one place, the open questions that arise when one surveys the latest developments. We hope that the discussion will stimulate more investigations toward promising approaches to overcoming therapy resistance and the infiltration of astrocytoma tumors within the brain.

## Data Availability

Not applicable.
